# Association of Cardiopulmonary Hemodynamics and Mortality in Veterans With Liver Cirrhosis: A Retrospective Cohort Study

**DOI:** 10.1161/JAHA.123.033847

**Published:** 2024-04-03

**Authors:** Arun Jose, Natalia Rahman, Alexander R. Opotowsky, Thomas J. Glorioso, Stephen W. Waldo, Katarina Zeder, Arnold Seto, Jean M. Elwing, Francis X. McCormack, Bradley A. Maron

**Affiliations:** ^1^ Veterans Affairs Cincinnati Healthcare System Cincinnati OH; ^2^ Rocky Mountain Regional VA Medical Center Aurora CO; ^3^ Veterans Affairs Boston Healthcare System Boston MA; ^4^ Brigham and Women’s Hospital and Harvard Medical School Boston MA; ^5^ Department of Medicine University of Maryland School of Medicine Baltimore MD; ^6^ The University of Maryland‐Institute for Health Computing Bethesda MD; ^7^ Heart Institute, Cincinnati Children’s Hospital Medical Center, University of Cincinnati OH; ^8^ Veteran’s Affairs Long Beach Healthcare System Long Beach CA; ^9^ University of Cincinnati OH; ^10^ Denver Research Institute Aurora CO; ^11^ CART Program, Office of Quality and Patient Safety, Veterans Health Administration Washington DC; ^12^ University of Colorado School of Medicine Aurora CO; ^13^ Ludwig Boltzmann Institute for Lung Vascular Research, Medical University of Graz Austria

**Keywords:** definition, liver disease, pulmonary hypertension, survival, Epidemiology, Pulmonary Hypertension, Mortality/Survival

## Abstract

**Background:**

Portopulmonary hypertension (PoPH), associated with increased mortality, can limit treatment options for liver diseases. Data on the continuum of clinical risk related to cardiopulmonary hemodynamics in PoPH are lacking.

**Methods and Results:**

As part of the United States national Veterans Affairs Clinical Assessment, Reporting, and Tracking database, we performed a retrospective cohort study of adults with cirrhosis undergoing right heart catheterization between October 1, 2017, and September 30, 2022. Pulmonary hypertension (mean pulmonary arterial pressure [mPAP] >20 mm Hg without PoPH) and PoPH (mPAP >20 mm Hg+pulmonary artery wedge pressure ≤15 mm Hg+pulmonary vascular resistance ≥3 WU) were defined by right heart catheterization hemodynamics. Multivariable Cox proportional hazards using natural splines for hemodynamic variables were used to evaluate the association between cardiopulmonary hemodynamics and mortality following right heart catheterization. A total of 4409 patients were included in the final analysis, predominantly men (96.3%), with a mean age of 68.5 years. Pulmonary hypertension and PoPH were observed in 71.6% and 10.2% of the cohort, respectively. Compared with a reference cardiac index of 2.5 L/min per m^2^, the hazard for mortality increased progressively with decreasing cardiac index, even after adjustment for mPAP and pulmonary vascular resistance. The minority of patients with PoPH (N=65, 14.5%) were prescribed pulmonary vasodilator therapy.

**Conclusions:**

These data suggest that pulmonary hypertension and PoPH are prevalent in veterans with chronic liver disease, but low use of targeted PoPH therapy persists. Cardiac function discriminated mortality risk across a wide range of mPAP and pulmonary vascular resistance values and may diagnose and clarify prognosis in this patient population.

**Nonstandard Abbreviations and Acronyms**
mPAP: mean pulmonary artery pressure
PAH: pulmonary arterial hypertension
PAWP: pulmonary artery wedge pressure
PH: pulmonary hypertension
PoPH: portopulmonary hypertension
PVR: pulmonary vascular resistance
RHC: right heart catheterization
VA: Veterans Affairs

**Clinical Perspective**

**What Is New?**

- In this cohort study of veterans with liver cirrhosis, several cardiopulmonary hemodynamic variables (mean pulmonary arterial pressure, pulmonary vascular resistance, cardiac index) were associated with mortality.
- Mortality remained strongly dependent on cardiac index across a wide range of mean pulmonary artery pressure and pulmonary vascular resistance values, with mortality increasing progressively at cardiac index <2.5 L/min per m^2^.
- A minority of portopulmonary hypertension patients eligible for pulmonary vasodilator therapy based on right heart catheterization hemodynamics were actually prescribed such treatments.

**What Are the Clinical Implications?**

- Cardiac index is a principal hemodynamic criterion for prognosticating pulmonary hypertension in the chronic liver disease population.
- Low use of targeted therapy in veterans with portopulmonary hypertension may represent a modifiable care gap in this patient population.

Pulmonary hypertension (PH) is diagnosed by an elevated mean pulmonary artery pressure (mPAP) >20 mm Hg.^1^, ^2^, ^3^, ^4^, ^5^ From the 6th World Symposium on PH, pulmonary arterial hypertension (PAH) is defined as a PH subtype that includes elevated pulmonary vascular resistance (PVR) ≥3 Wood units (WU) in the absence of elevated pulmonary artery wedge pressure (PAWP≤15 mm Hg).^2^ In 2022, the PVR threshold used to diagnose PAH was lowered to ≥2 WU.^1^

These hemodynamic definitions for PAH have been extended to portopulmonary hypertension (PoPH), a somewhat unique subphenotype of PAH that occurs exclusively in patients with underlying portal hypertensive liver disease (typically in the context of liver cirrhosis). However, it is not known if the diagnostic and classification framework for PAH is optimal for patients with PoPH,^1^, ^2^, ^3^, ^4^, ^5^, ^6^, ^7^ and data on the spectrum of risk associated with hemodynamic parameters that are prognostic in other contexts (eg, mPAP, PVR, and cardiac index)^3^, ^7^, ^8^, ^9^ have failed to consistently predict survival in PoPH.^10^, ^11^, ^12^, ^13^, ^14^, ^15^, ^16^, ^17^, ^18^ Additionally, a hyperdynamic circulation is frequently appreciated in the management of patients with PoPH at point of care, which may confound the repurposing of cardiopulmonary hemodynamic ranges that are used to delineate clinical risk in patients with PAH to those with underlying liver disease.^19^, ^20^, ^21^ In order to clarify the association between cardiopulmonary hemodynamics and mortality in liver cirrhosis, we used the Veterans Affairs (VA) Clinical Assessment, Reporting, and Tracking Program, a national cohort of patients referred for right heart catheterization (RHC).^22^

## METHODS

Data supporting the findings of this study are available through the Corporate Data Warehouse from the US Veterans Administration and can be requested via the VA Informatics and Computing Infrastructure Workspace: (https://www.research.va.gov/programs/vinci/default.cfm).

### Data Sources

The VA Clinical Assessment, Reporting, and Tracking Program uses a software application embedded in the VA electronic health record for documentation of all invasive cardiovascular procedures performed in the national VA health care system, including assessment of hemodynamic measurements.^3^ This information is linked to the VA electronic health record to allow tracking of longitudinal outcomes for quality and safety purposes.^3^, ^7^, ^22^ Additional information on covariates and outcomes was derived from VA administrative data sources.

### Study Population

We evaluated all adult patients ≥18 years old diagnosed with liver cirrhosis undergoing RHC between October 1, 2017, and September 30, 2022. For patients undergoing multiple RHC procedures during the study period, only data from the earliest qualifying study were analyzed. This study was reviewed and approved by the Colorado Multiple Institutional Review Board, with a waiver of informed consent.

A diagnosis of liver cirrhosis was based on a combination of *International Classification of Diseases, Tenth Revision* (*ICD‐10*) diagnostic codes and laboratory values.^23^, ^24^ Patients had to meet ≥2 of the following criteria: a diagnostic code indicating underlying liver disease, a diagnostic code indicating portal hypertensive liver disease sequelae, or evidence of liver dysfunction on laboratory testing. The full list of qualifying *ICD‐10* codes and laboratory values can be found in Data S1. Patients with a prior history of liver transplantation, and those with diagnostic codes indicating hepatopulmonary syndrome, were excluded from this study. Subjects with missing data were excluded.

### Covariates

Hemodynamic variables including pulmonary arterial systolic and diastolic pressures, mPAP, PAWP, cardiac output via estimated Fick or thermodilution methods, and PVR were collected through the Clinical Assessment, Reporting, and Tracking application. Full details on hemodynamic variables in the Clinical Assessment, Reporting, and Tracking application can be found in Data S1. In this study, for the purposes of labeling simplicity, PH was defined based on RHC data as mPAP>20 mm Hg.^1^, ^2^ PoPH was defined based on RHC data as PH (mPAP>20 mm Hg) with PAWP≤15 mm Hg and PVR of ≥3 WU, based on the 6th World Symposium on Pulmonary Hypertension guidelines.^2^ Similarly, postcapillary PH was defined based on RHC data as PH (mPAP>20 mm Hg) with an elevated PAWP (>15 mm Hg). We used the term “hyperdynamic circulation” in reference to patients with PH (mPAP>20 mm Hg) but without elevated PAWP (≤15 mm Hg) or PVR (<3 WU).

Additional clinical data were extracted from the VA electronic health record, including demographics (age, sex, race, ethnicity), outpatient medical therapy, laboratory information (within 6 months of RHC), and comorbid medical conditions (documented via *ICD‐10* codes in the 2 years preceding RHC), as described previously.^22^ Race and ethnicity data recorded in the VA electronic health record are collected through self‐identification either at VA benefits enrollment or during an inpatient or outpatient clinical encounter. Detailed information on how specific comorbid medical conditions were defined can be found in Data S1.

Targeted therapy for PAH (ie, pulmonary vasodilators) was defined as an outpatient prescription fill of an endothelin receptor antagonist, soluble guanylate cyclase stimulator, prostacyclin pathway modulator, or phosphodiesterase‐5 inhibitor within 90 days following RHC. Supply of phosphodiesterase‐5 inhibitors therapy for erectile dysfunction is restricted in the Veterans Administration system to 4 doses per month, allowing us to identify and exclude these prescriptions from the analysis.^25^ Outpatient prescription fill of systemic anticoagulation, beta‐adrenergic receptor antagonist therapy, and liver cirrhosis therapy were also captured within the 90 days before RHC. Full details on medication definitions in this study can be found in Data S1.

Laboratory information within 6 months before RHC included serum creatinine, bilirubin, sodium, and international normalized ratio. The Model for End‐Stage Liver Disease score (MELD), sodium‐adjusted MELD, and MELD excluding international normalized ratio (MELD‐XI) were calculated in the standard fashion.^26^, ^27^ Full details on MELD calculations can be found in Data S1.

### Exposures

The primary outcome in this study was time to death (all‐cause mortality) following RHC.^28^ Censoring events were either (1) end of study follow‐up or (2) liver transplantation. Patients were followed for up to 3 years following RHC, until a mortality event occurred, a liver transplantation occurred, or until September 30, 2022, whichever occurred earliest.

### Statistical Analysis

Differences between groups were compared using a multiple degrees of freedom chi‐square test or a 1‐way ANOVA for categorical or continuous variables, respectively. Results are presented as mean (SD) or frequency (%) for continuous and categorical variables, respectively. Estimated Kaplan–Meier curves were used to compare both 6‐month, 1‐year, and 3‐year survival rates between disease subtype groups (non‐PH cirrhosis, PH cirrhosis, and PoPH) and 3‐year survival rates with or without the use of PAH targeted therapy in the group with PoPH. To model the association between hemodynamic variables and time to event (mortality), Cox proportional hazards regression models accounting for clustering by RHC site (ie, frailty models with robust SEs) were constructed separately using natural splines for mPAP, PVR, PAWP, and cardiac index. Models were censored for liver transplantation. An optimum number of knots were determined for each hemodynamic parameter using the Akaike information criterion. Models for each hemodynamic variable (mPAP, PVR, PAWP, and cardiac index) were adjusted for MELD‐XI, comorbidities, and demographics (age, sex, race, and ethnicity). An additional model for cardiac index was further adjusted for mPAP and PAWP.

Hazard ratio (HR) estimates were calculated relative to reference values. The reference levels for mPAP (10 mm Hg)^1^, ^29^ and PVR (1 WU)^7^ were based on expected hemodynamic parameter values from healthy subjects undergoing RHC, and the reference value for cardiac index (2.5 L/min per m^2^)^1^, ^2^, ^30^ was based on a clinically accepted and evidence‐based cutoff for impairment. The hazard curves were plotted for the adjusted models using 1‐year and 3‐year outcomes.

We also dichotomized the cohort by MELD‐XI scores of ≥12 versus <12, based on a clinically accepted and evidence‐based cutoff for mortality risk.^31^, ^32^ For each MELD‐XI group (≥12 and <12), Cox proportional hazards regression models accounting for clustering by RHC site (ie, frailty models with robust SEs) were constructed separately using natural splines for mPAP, PVR, PAWP, and cardiac index. Models were censored for liver transplantation. An optimum number of knots was determined for each hemodynamic parameter using the Akaike information criterion. For each MELD‐XI group (≥12 and <12), models for each hemodynamic variable (mPAP, PVR, PAWP, and cardiac index) were adjusted for comorbidities and demographics (age, sex, race, and ethnicity).

All statistical analyses were performed in R version 4.2.1 (R Foundation for Statistical Computing, Vienna, Austria). A 2‐sided *P* value of <0.05 was considered statistically significant. One author (A. J.) takes full responsibility for the data analysis and data integrity.

## RESULTS

### Clinical Profile

**Figure 1:**
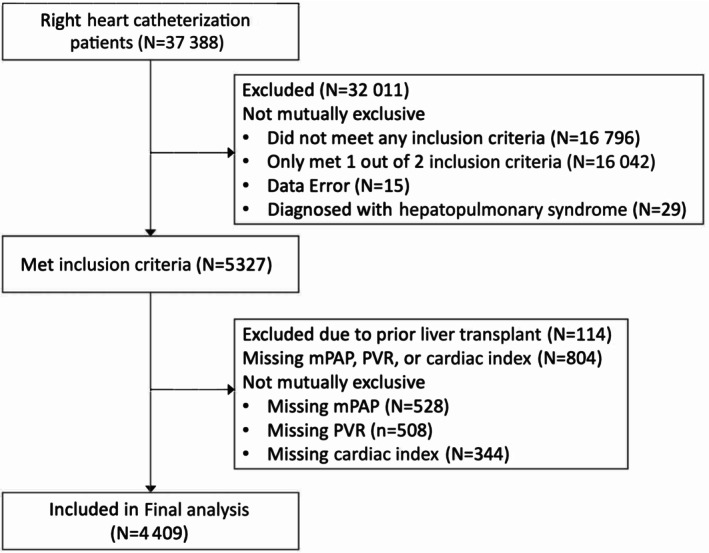
Consolidated Standards of Reporting Trials diagram for patient throughput in the analysis of this study. mPAP indicates mean pulmonary arterial pressure; and PVR, pulmonary vascular resistance.

A total of 4409 patients met eligibility criteria and were included in the final analysis (Figure 1). Demographic and clinical characteristics of the cohort, stratified by PH type, are listed in the Table. The overall distributions of hemodynamic values are presented in Figure S1 and Table S1. The prevalence of PoPH was 10.2% (N=449) in this cohort, with 71.6% (N=3158) having PH but not meeting the full criteria for PoPH (“PH cirrhosis,” either hyperdynamic circulation [N=492, 15.6%] or postcapillary PH [N=2666, 84.4%]). Almost one‐fifth of the cohort (N=802, 18.2%) were patients with cirrhosis without PH (“non‐PH cirrhosis”). Lowering the PVR threshold for diagnosing PoPH to 2 WU (mPAP>20 mm Hg+PAWP≤15 mm Hg+PVR>2 WU)^1^ captured an additional 209 patients, increasing PoPH prevalence to 14.9% (N=658). By comparison, using the “classical” definitions for PH (mPAP≥25 mm Hg) and PoPH (mPAP≥25 mm Hg+PAWP≤15 mm Hg+PVR≥3WU) captured 15.3% (N=482 of 3158) and 16.3% (N=73 of 449) fewer patients with these diagnoses, respectively, yielding a PH cirrhosis prevalence of 60.7% (N=2676) and a PoPH prevalence of 8.5% (N=376).

**Table 1:** Demographic and Clinical Characteristics of Patients Stratified by Pulmonary Hypertension Status

	Non‐PH cirrhosis (N=802)	Hyperdynamic circulation (N=492)	Postcapillary PH (N=2666)	PoPH (N=449)	*P* value
Demographics					
Age, y	67.6 (9.7)	68.6 (9.1)	68.6 (9.0)	69.3 (8.3)	0.01
Sex, male	764 (95.3)	467 (94.9)	2585 (97.0)	430 (95.8)	0.03
Race					<0.01
American Indian or Alaska Native	7 (0.9)	2 (0.4)	24 (0.9)	2 (0.4)	
Asian	3 (0.4)	5 (1.0)	10 (0.4)	8 (1.8)	
Black	180 (22.4)	132 (26.8)	787 (29.5)	184 (41.0)	
Native Hawaiian or Other Pacific Islander	5 (0.6)	4 (0.8)	28 (1.1)	4 (0.9)	
Unknown	37 (4.6)	23 (4.7)	95 (3.6)	28 (6.2)	
White	570 (71.1)	326 (66.3)	1722 (64.6)	223 (49.7)	
Laboratory values					
Bilirubin, mg/dL	1.4 (1.2)	1.5 (2.9)	1.5 (1.7)	1.3 (0.9)	0.11
Creatinine, mg/dL	1.6 (1.4)	1.9 (1.8)	2.1 (1.7)	1.9 (1.6)	<0.01
MELD adjusted for serum sodium	13.2 (5.8)	14.7 (6.6)	16.9 (7.0)	15.4 (6.6)	<0.01
MELD excluding international normalized ratio	13.7 (5.7)	15.3 (6.8)	16.8 (6.9)	15.4 (6.6)	<0.01
Ethnicity					0.14
Hispanic or Latino	53 (6.6)	24 (4.9)	139 (5.2)	32 (7.1)	
Not Hispanic or Latino	731 (91.1)	453 (92.1)	2483 (93.1)	409 (91.1)	
Unknown	18 (2.2)	15 (3.0)	44 (1.7)	8 (1.8)	
Hemodynamic variables					
Mean pulmonary arterial pressure, mm Hg	16.1 (3.2)	24.4 (3.3)	35.0 (7.2)	32.2 (7.5)	<0.01
Pulmonary vascular resistance, WU	1.3 (0.9)	1.9 (0.6)	2.4 (1.6)	5.0 (2.1)	<0.01
Cardiac index, L/min per m^2^	2.7 (0.9)	3.0 (0.9)	2.4 (0.9)	2.2 (0.6)	<0.01
Pulmonary artery wedge pressure, mm Hg	9.4 (4.0)	12.7 (2.4)	23.9 (5.8)	10.8 (3.4)	<0.01
Proportion with cardiac index <2.5 L/min per m^2^	368 (45.9)	154 (31.3)	1620 (60.8)	326 (72.6)	<0.001
Diabetes	426 (53.1)	310 (63.0)	1727 (64.8)	265 (59.0)	<0.01
Systemic hypertension	738 (92.0)	466 (94.7)	2560 (96.0)	421 (93.8)	<0.01
Chronic kidney disease	331 (41.3)	267 (54.3)	1696 (63.6)	260 (57.9)	<0.01
Chronic obstructive pulmonary disease	298 (37.2)	231 (47.0)	1379 (51.7)	267 (59.5)	<0.01
Sickle cell anemia	2 (0.2)	7 (1.4)	9 (0.3)	2 (0.4)	<0.01
Pulmonary embolism	50 (6.2)	35 (7.1)	152 (5.7)	36 (8.0)	0.22
Interstitial lung disease	36 (4.5)	32 (6.5)	103 (3.9)	63 (14.0)	<0.01
Connective tissue disease	42 (5.2)	24 (4.9)	106 (4.0)	29 (6.5)	0.08
Coronary artery disease	467 (58.2)	320 (65.0)	1967 (73.8)	303 (67.5)	<0.01
Sleep apnea	327 (40.8)	273 (55.5)	1422 (53.3)	209 (46.5)	<0.01
Left heart failure	359 (44.8)	258 (52.4)	1685 (63.2)	248 (55.2)	<0.01
Outpatient prescription fill					
PAH targeted therapy	5 (0.6)	17 (3.5)	55 (2.1)	65 (14.5)	<0.01
Liver cirrhosis medication	85 (10.6)	29 (5.9)	113 (4.2)	23 (5.1)	<0.01
Systemic anticoagulation medication	289 (36.0)	214 (43.5)	1126 (42.2)	171 (38.1)	<0.01
Warfarin	22 (2.7)	16 (3.3)	107 (4.0)	20 (4.5)	0.23
Beta‐adrenergic receptor antagonist therapy	466 (58.1)	282 (57.3)	1685 (63.2)	252 (56.1)	<0.01
Cause of liver cirrhosis					
Liver cirrhosis (any type) or portal hypertension	751 (93.6)	450 (91.5)	2492 (93.5)	406 (90.4)	0.05
Viral cirrhosis	240 (29.9)	127 (25.8)	793 (29.7)	156 (34.7)	0.03
Nonalcoholic steatohepatitis	262 (32.7)	154 (31.3)	794 (29.8)	110 (24.5)	0.02
Alcoholic cirrhosis	184 (22.9)	87 (17.7)	436 (16.4)	64 (14.3)	<0.01
Esophageal varices, hepatic encephalopathy, or hepatorenal syndrome	297 (37.0)	171 (34.8)	765 (28.7)	145 (32.3)	<0.01
Laboratory evidence of liver dysfunction	746 (93.0)	461 (93.7)	2588 (97.1)	426 (94.9)	<0.01

Outpatient prescription fill of PAH targeted therapy included endothelin receptor antagonists (bosentan, ambrisentan, macitentan), soluble guanylate cyclase stimulators (riociguat), prostacyclin pathway modulators (selexipag, epoprostenol, treprostinil), or phosphodiesterase‐5 inhibitors (sildenafil, tadalafil). Outpatient prescription fill of liver cirrhosis medication included rifaximin, propranolol, nadolol, lactulose, or ursodiol. Outpatient prescription fill of systemic anticoagulation medication included warfarin, heparin, rivaroxaban, apixiban, edoxaban, dabigatran, or betrixaban. Outpatient fill of beta‐adrenergic receptor antagonist therapy included carvedilol, propranolol, bisoprolol, labetalol, and metoprolol. Data are presented as mean (SD) for continuous variables and N (%) for categorical variables. *P* value calculated based on a multiple degrees of freedom chi‐square test or a 1‐way ANOVA for categorical or continuous variables, respectively. MELD indicates model for end‐stage liver disease score; PAH, pulmonary arterial hypertension; PH, pulmonary hypertension; and PoPH, portopulmonary hypertension.

There was no significant difference in Hispanic or Latino ethnicity between groups for subjects with hyperdynamic circulation, postcapillary PH, non‐PH cirrhosis, and PoPH. Patients with postcapillary PH were more likely to be male as compared with groups with PoPH, hyperdynamic circulation, and non‐PH cirrhosis. Subjects with PoPH, when compared with the groups with non‐PH cirrhosis, hyperdynamic circulation, and postcapillary PH, were more likely to be older (69.3 [8.3] versus 67.6 [9.7] versus. 68.6 [9.1] versus 68.6 [9.0], *P*<0.01), self‐reported as Black race (41.0% versus 22.4% versus 26.8% versus 29.5%, *P*<0.01), diagnosed with interstitial lung disease (14.0% versus 4.5% versus 6.5% versus 3.9%, *P*<0.01) or chronic obstructive pulmonary disease (59.5% versus 37.2% versus 47.0% versus 51.7%, *P*<0.01), and have been prescribed PAH therapy (14.5% versus 0.6% versus 3.5% versus 2.1%, *P*<0.01). A low cardiac index (<2.5 L/min per m^2^) was significantly more prevalent in the group with PoPH as compared with the groups with non‐PH cirrhosis, hyperdynamic circulation, and postcapillary PH (72.6% versus 45.9% versus 31.3% versus 60.8%, *P*<0.01). The group with postcapillary PH had higher observed rates of medical comorbidities known to increase PH risk (eg, chronic kidney disease, diabetes, systemic hypertension, coronary artery disease, and left heart failure), greater outpatient prescription fills of beta‐adrenergic receptor antagonist therapy as well as higher observed sodium‐adjusted MELD (16.9 [7.0] versus 15.4 [6.6] versus 13.2 [5.8] versus 14.7 [6.6], *P*<0.01) and MELD‐XI (16.8 [6.9] versus 15.4 [6.6] versus 13.7 [5.7] versus 15.3 [6.8], *P*<0.01) scores as compared with the groups with PoPH, hyperdynamic circulation, and non‐PH cirrhosis, respectively.

When compared with the groups with PoPH, postcapillary PH, and non‐PH cirrhosis, patients with hyperdynamic circulation were more likely to carry diagnoses of sickle cell disease and sleep apnea and be prescribed systemic anticoagulation medication. There was no significant difference between groups in the use of warfarin at the time of RHC. Patients with non‐PH cirrhosis were more likely to carry diagnoses of alcoholic liver cirrhosis and nonalcoholic steatohepatitis as compared with the groups with PoPH, postcapillary PH, and hyperdynamic circulation. Patients with PoPH were more likely to have cirrhosis secondary to viral hepatitis compared with the groups with non‐PH cirrhosis, postcapillary PH, and hyperdynamic circulation.

### Cardiopulmonary Hemodynamics

**Figure 2:**
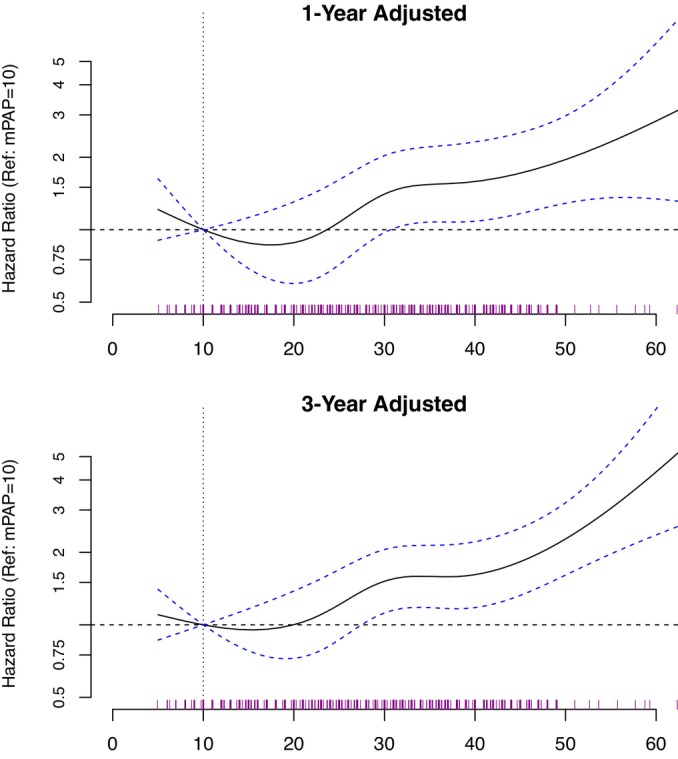
The association between mPAP and mortality in the study cohort. Adjusted hazard ratio for all‐cause mortality at 1 and 3 years following right heart catheterization as a function of mPAP. Hazard ratios (solid line) with 95% CIs (dashed bands) are plotted for mPAP relative to a reference value of 10 mm Hg (vertical dotted line). The hazard ratios on the y‐axis are demarcated by a logarithmic scale, and a hazard ratio of 1 is depicted by a horizontal dotted line. A rugplot showing the distribution of mPAP values is displayed directly above the x‐axis. Multivariable models adjusted for age, sex, race, ethnicity, Veterans Administration procedural site, MELD‐XI, and comorbidities. MELD‐XI indicates model for end‐stage liver disease excluding international normalized ratio; and mPAP, mean pulmonary arterial pressure.

We focused on mPAP, PVR, PAWP, and cardiac index in our outcome analyses as these variables are used in clinical practice to diagnose and risk‐stratify PoPH. When compared with a reference mPAP of 10 mm Hg, we observed a continuous rise in adjusted hazard for 1‐year mortality beginning at mPAP of approximately 32 mm Hg (HR, 1.49 [95% CI, 1.04–2.14], *P*=0.03) and 3‐year mortality at mPAP of approximately 28 mm Hg (HR, 1.41 [95% CI, 1.03–1.93], *P*=0.03) (Figure 2, Table S2). Hazard for mortality also increased continuously at PVR values above the reference of 1 WU (Figure S2). At a PVR of 3 WU, the 1‐year adjusted HR for mortality was 1.24 (95% CI, 1.12–1.39, *P*<0.01) and the 3‐year adjusted HR for mortality was 1.22 (95% CI, 1.12–1.34, *P*<0.01). When compared with a reference PAWP of 10 mm Hg, we observed a continuous rise in adjusted hazard for mortality beginning at a PAWP of approximately 15 mm Hg, with a 1‐year adjusted HR for mortality of 1.13 (95% CI, 1.01–1.26, *P*=0.03) and a 3‐year adjusted HR for mortality of 1.15 (95% CI, 1.05–1.26, *P*<0.01) (Table S2, Figure S3).

**Figure 3:**
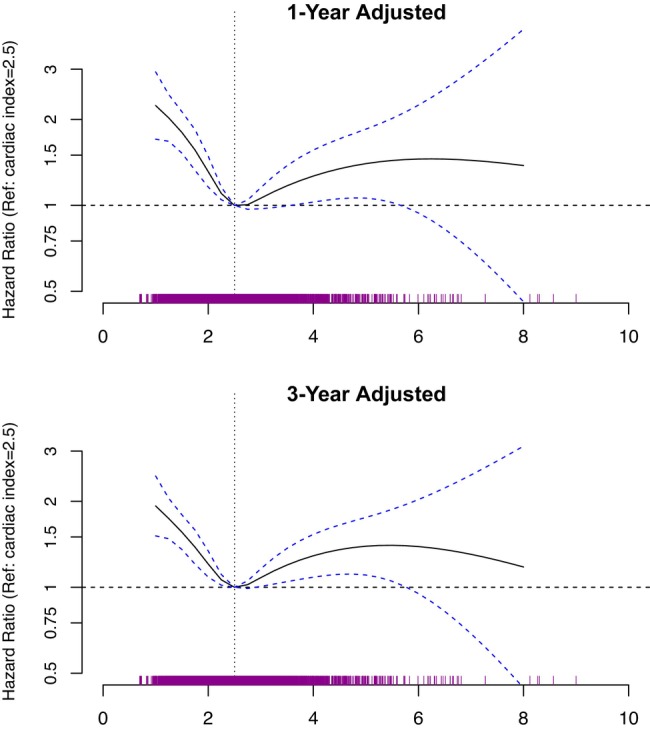
The association between cardiac index and mortality in the study cohort. Adjusted hazard ratio for all‐cause mortality at 1 and 3 years following right heart catheterization as a function of cardiac index. Hazard ratios (solid line) with 95% CIs (dashed bands) are plotted for mPAP relative to a reference value of 2.5 L/min per m^2^ (vertical dotted line). The hazard ratios on the y‐axis are demarcated by a logarithmic scale, and a hazard ratio of 1 is depicted by a horizontal dotted line. A rugplot showing the distribution of cardiac index values is displayed directly above the x‐axis. Multivariable models adjusted for age, sex, race, ethnicity, Veterans Administration procedural site, MELD‐XI, and comorbidities. MELD‐XI indicates model for end‐stage liver disease excluding international normalized ratio; and mPAP, mean pulmonary arterial pressure.

The 1‐year unadjusted mortality risk for patients with cardiac index <2.5 L/min per m^2^ was significantly greater than patients with cardiac index ≥2.5 L/min per m^2^ (31% versus 28%, *P*=0.02) (Figure S4). Hazard for mortality increased progressively beginning at levels below the reference of 2.5 L/min per m^2^; at a cardiac index of 2.0 L/min per m^2^, the 1‐year adjusted HR for mortality was 1.31 (95% CI, 1.16–1.49), and the 3‐year adjusted HR for mortality was 1.21 (95% CI, 1.09–1.34) (Figure 3, Table S3). A directionally similar and statistically significant relationship between cardiac index and mortality was maintained even after adjusting for mPAP and PAWP (Table S4). When compared with unadjusted models, adjustment for MELD‐XI, comorbidities, Veterans Administration procedural site, and demographics (age, sex, race, and ethnicity) did not substantially affect the association between cardiopulmonary hemodynamic measurements (mPAP, PVR, PAWP, cardiac index) and mortality risk (Table S5).

We next dichotomized the cohort by MELD‐XI scores of ≥12 versus <12. In patients with MELD‐XI scores ≥12, we observed a continuous rise in adjusted hazard for both 1‐year and 3‐year mortality beginning at mPAP of approximately 30 mm Hg (1‐year mortality HR, 1.65 [95% CI, 1.04–2.61], *P*=0.03; 3‐year mortality HR, 1.92 [95% CI, 1.29–2.87], *P*<0.01) and a PAWP of approximately 15 mm Hg (1‐year mortality HR, 1.12 [95% CI, 1.01–1.24], *P*=0.03; 3‐year mortality HR, 1.13 [95% CI, 1.03–1.24], *P*<0.01) (Table S6). In the MELD‐XI scores ≥12 group, hazard for mortality also increased at PVR values above the reference of 1 WU, and at cardiac index values below the reference of 2.5 L/min per m^2^. In the MELD‐XI <12 group, elevated mPAP >28 mm Hg, PAWP >12 mm Hg, and PVR >1 WU were not associated with increased hazard for mortality (Table S7). A significant (albeit attenuated) relationship between cardiac index and mortality remained in the MELD‐XI <12 group: cardiac index of 1.5 L/min per m^2^ was associated with a 1‐year adjusted HR for mortality of 1.76 (95% CI, 1.18–2.62) and a 3‐year adjusted HR for mortality of 1.52 (95% CI, 1.12–2.07).

**Figure 4:**
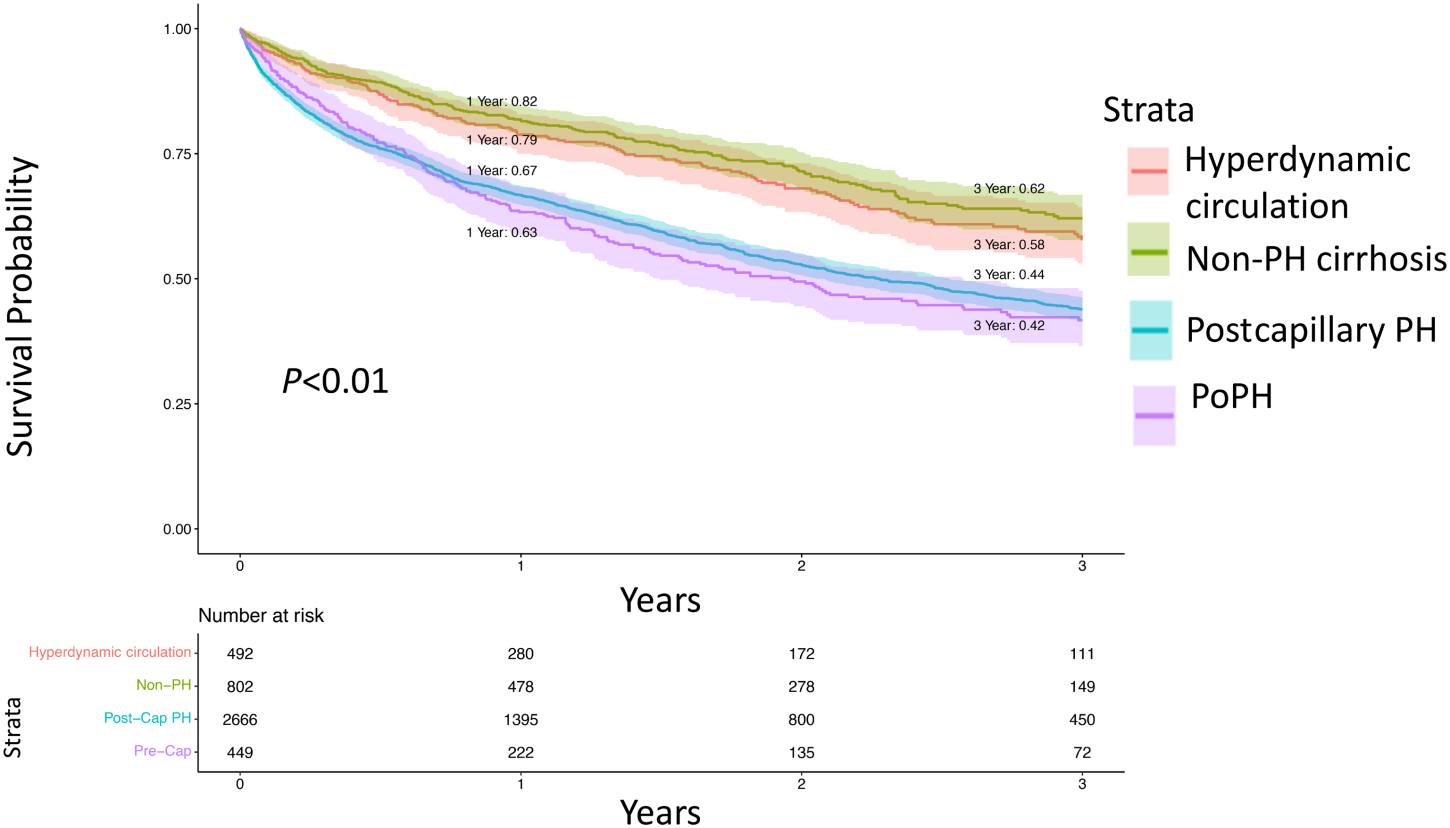
Kaplan–Meier analysis for survival probability stratified by PH phenotype. Kaplan–Meier analysis for the patient cohort stratified by PH phenotype. Red, hyperdynamic circulation; green, non‐PH cirrhosis; blue, postcapillary PH; purple, PoPH. Log‐rank test *P* value displayed. PH indicates pulmonary hypertension; and PoPH, portopulmonary hypertension.

We next estimated unadjusted Kaplan–Meier curves for all‐cause mortality (Figure 4, Table S8) stratified by the subgroups with hyperdynamic circulation, non‐PH cirrhosis, postcapillary PH, and PoPH. Mortality rates at either 1 or 3 years following RHC were significantly greater in the group with postcapillary PH relative to the groups with non‐PH cirrhosis (1‐year mortality 33% versus 18%; 3‐year mortality 56% versus 38%; *P*<0.01 for all risk difference comparisons) and hyperdynamic circulation (1‐year mortality 33% versus 21%; 3‐year mortality 56% versus 42%; *P*<0.01 for all risk difference comparisons). Mortality rates at either 1 or 3 years following RHC were also significantly greater in patients with PoPH relative to the groups with non‐PH cirrhosis (1‐year mortality 37% versus 18%; 3‐year mortality 58% versus 38%; *P*<0.01 for all risk difference comparisons) and hyperdynamic circulation (1‐year mortality 37% versus 21%; 3‐year mortality 58% versus 42%; *P*<0.01 for all risk difference comparisons). Mortality rates for patients with PoPH were greater than those for patients with postcapillary PH at all time points, but this difference was not statistically significant. Focusing on patients with PoPH, the use of PAH targeted therapy (N=65, 14.5%) did not have a significant effect on survival over time (Figure S5). When restricting the analysis to the “classical” definition of PoPH, the proportion of patients with PoPH prescribed PAH‐targeted therapy (N=62, 16.5%) was only marginally different, and a significant association between treatment and survival did not emerge.

## DISCUSSION

Data from this study in a national cohort of veterans referred for RHC establish the risk profile of PoPH and provide new insight on the prognostic relevance of cardiopulmonary hemodynamics in patients with liver disease. Specifically, our findings showed that the current mPAP threshold of >20 mm Hg used to define PH was highly prevalent but that elevated clinical risk related to mPAP is evident beginning with mPAP >28 mm Hg. In turn, this study redirects emphasis to central cardiac function when prognosticating patients with PoPH, because a discrete PVR range that identifies risk onset was not observed, whereas low cardiac index (<2.5 L/min per m^2^) discriminated mortality risk significantly. A potential gap in clinical care emerged from the current study as the administration of PAH therapy was uncommon despite patient eligibility in accordance with guideline‐based recommendations for treatment. Taken together, results from this study clarify the diagnostic framework for PoPH, emphasizing the clinical relevance of low cardiac index in this cohort, and suggest that opportunity may exist to improve the dissemination of medical therapy to at‐risk patients.

The prevalence of PH and PoPH in this study was considerably higher compared with nonveteran populations, maintained even when a conservative mPAP threshold (>25 mm Hg) was used to classify patients.^33^, ^34^, ^35^, ^36^, ^37^ For example, in a prospective study of 1235 nonveteran patients with Child–Pugh class B or C liver dysfunction referred for RHC, mPAP>20 mm Hg was identified in only 7.9% of subjects, and “classically” defined PoPH in 5.3% of subjects.^33^ Similarly, 2 European studies of candidates for liver transplant referred for RHC testing identified PoPH in 4.6% to 4.8% of subjects.^34^, ^35^ The population in the current study was generally older, male, and characterized by medical comorbidities that predispose to PH independent of underlying liver disease, which may have contributed to the observed prevalence of PH and PoPH. Nevertheless, data from this study establish veterans with chronic liver disease as a population of patients predisposed to PoPH. Consequently, the presence of liver disease in veterans may constitute a subgroup for whom more intensive PoPH screening is warranted to facilitate earlier diagnosis, which itself is an emerging priority in the field of clinical pulmonary vascular disease.^38^

We observed that PH was associated with increased mortality in patients with chronic liver disease. However, modeling mPAP as a continuous variable clarified that this clinical risk is largely observed when mPAP >28 mm Hg. A hyperdynamic circulation is part of the PoPH syndrome^19^, ^20^, ^21^; thus, this observation is consistent with the flow‐mediated elevation in mPAP originally described by Paul Wood^39^ and is directly in line with the rationale proposed by Simmoneu and colleagues to include PVR as a determinant of pulmonary vascular disease.^2^ Arbitrating the appropriate threshold for PVR in hyperdynamic forms of PH is challenging, however, because PVR is inversely proportional to cardiac index. Indeed, we observed that clinical risk in PoPH is evident throughout the PVR spectrum, making identification of a precise cutoff with clinical and prognostic value in liver disease challenging. Thus, PVR remains relevant when prognosticating patients, but our data also suggest that central cardiac function (ie, cardiac index, from which PVR is derived) could be particularly informative in prognosticating patients with PoPH and liver disease.

Our finding emphasizing the clinical importance of low cardiac index (<2.5 L/min per m^2^) in PoPH is consistent with other large retrospective analyses in which this association has also been observed. In a multivariable analysis of the French national PH registry, cardiac index was the only hemodynamic variable predictive of PoPH survival, and an analysis of the UK national PH registry demonstrated that patients with PoPH with a higher cardiac index had significantly better survival than those with a lower cardiac index.^15^, ^16^ Even in the high‐risk clinical scenario of liver transplantation, evidence suggests that mPAP is less important than right ventricular function in predicting posttransplant outcomes.^40^, ^41^ Notably, the majority of patients in our cohort fell into this high‐risk group, including almost half of patients without PH. Although cardiovascular dysfunction occurs frequently in cirrhosis, and cirrhotic cardiomyopathy is believed to be present in half of all patients with end‐stage liver disease, we were unable to determine if the impaired cardiac function seen in our study population was reflective of this established link between liver and cardiovascular disease, a consequence of the high rate of medical comorbidities present in our study population, a marker of underlying pulmonary vascular disease risk, or some combination thereof.^42^, ^43^ Our analyses aimed to clarify this relationship by adjusting for cardiopulmonary vascular hemodynamics (mPAP, PAWP), important comorbid conditions (eg, left heart failure), and concurrent medications (eg, beta‐adrenergic receptor antagonist therapy) when analyzing clinical risk in a subgroup of patients with compensated liver disease (as defined by MELD‐XI <12). However, the relationship between impaired cardiac function (namely low cardiac index) and increased mortality in patients with liver disease persisted. In particular, we observed that survival of patients with PoPH was strongly linked to cardiac function across a wide range of mPAP values, which was maintained after adjusting for potential confounding variables, and low cardiac index was the only hemodynamic variable associated with a significantly increased hazard for mortality in patients with compensated liver disease (MELD‐XI <12). In light of these results, it may be the case that considering cardiac index per se is helpful for clarifying diagnostic criteria for patients with PH and PoPH with chronic liver disease, thereby setting the stage for future studies that aim to contemporize the suitability of goal‐directed therapy and liver transplantation in PoPH.^10^, ^44^

We found that a minority of patients with PoPH eligible for PAH therapy were, in fact, prescribed treatment. This finding was evident even though PoPH was particularly high risk in this cohort, with observed survival far less than what has been reported in French and UK PoPH registry studies.^1^, ^2^, ^15^, ^16^ Other VA studies have shown that although many patients with PAH frequently experience delays in starting targeted therapy, patients with PoPH in particular are less likely to receive aggressive therapy compared with patients with idiopathic PAH.^45^, ^46^ Furthermore, only 1 randomized clinical trial dedicated to the treatment of PoPH has been published,^47^ and the safety profile of PAH‐targeted therapy use in patients with liver disease remains unresolved. Taken together, these findings emphasize awareness and the need for additional prospective data to contemporize guidance on the management of patients with PoPH toward improving survival.

### Limitations

There are several limitations that should be considered when interpreting our findings. First, this is a retrospective study involving a population of mostly male US veteran patients referred for RHC; thus, our findings may not be generalizable to other patient populations. We used a combination of *ICD‐10* codes and laboratory values to identify patients with portal hypertension, but confirmation via direct portal pressure measurements was not available and alternative reasons for fulfilling diagnostic criteria other than portal hypertensive liver disease are possible.^23^, ^24^, ^25^, ^26^, ^27^, ^28^, ^29^, ^30^, ^31^, ^32^, ^33^, ^34^, ^35^, ^36^ We assessed medication usage within 90 days following RHC testing using a validated methodology,^25^, ^48^ but this approach may nonetheless have missed some patients who were initiated on targeted therapy beyond our 90‐day time point or received medication from entities outside of the VA system.

## CONCLUSIONS

This analysis demonstrates a considerable burden of PH cirrhosis and PoPH among the veteran population, with mortality strongly dependent on central cardiac function across a wide range of mPAP and PVR values. Findings from this study suggest in particular that cardiac index <2.5 L/min per m^2^ per se is a principal hemodynamic criterion to prognosticate PoPH in the population with chronic liver disease, paving the way for a disease‐specific diagnostic framework in at‐risk patients. Low use of targeted PAH therapy in patients with PoPH also emerged as a central finding in this study and may indicate a modifiable care gap. Data from this study warrant prospective studies that aim to optimize detection of PH cirrhosis and PoPH systematically as well as clinical trials focused on interventions that ameliorate excess burden of mortality associated with this high‐risk population.

## Sources of Funding

This work is supported by a grant from the U. S. Department of Veterans Affairs (CART Award #R26) (AJ and FXM). This work is also supported by Francis Family Foundation (Parker B. Francis Fellowship) (A.J.) and NIH/ National Heart, Lung, and Blood Institute (NHLBI) grant (1K23HL164971‐01A1) (A.J.). This work is also supported by MPowering the State, a formal collaboration between the University of Maryland, College Park, and the University of Maryland, Baltimore. The views expressed in this article are those of the authors and do not necessarily reflect the position or policy of the Department of Veterans Affairs or the United States government.

## Disclosures

Dr Jose has reports investigator‐initiated research supported by the United Therapeutics corporation. Dr Opotowsky has served on the consultant or advisory board of Janssen Pharmaceuticals. Dr Elwing has received research/grant support from Janssen, United Therapeutics, Liquidia, Phase Bio, Goassamer Bio, Bayer, Merck, Altavant, Aerovate, Tenax, and Pharmosa, and serves on the consultant or advisory board of United Therapeutics, Altavant, Aerovate, Bayer, Goassamer Bio, Liquidia, Merck, and Janssen. Dr Maron reports investigator‐initiated research supported by the Boston Biomedical Innovation Center and Deerfield Company (outside the scope of this project), and serves on the consultant or advisory board of Janssen Pharmaceuticals, Tenax, and Regeneron. The remaining authors have no disclosures to report.

## Supporting information

Data S1
Tables S1–S8
Figures S1–S5
