# Supplementary material for: Association of Cardiopulmonary Hemodynamics and Mortality in Veterans With Liver Cirrhosis: A Retrospective Cohort Study
Source: J Am Heart Assoc. 2024 Apr 3;13(8):e033847. doi: 10.1161/JAHA.123.033847 (PMC11262483; doi:10.1161/JAHA.123.033847)
Supplement: Supplementary file 1 — Data S1 Tables S1–S8 Figures S1–S5 [file JAH3-13-e033847-s001.pdf]

# Supplemental Material

## Data S1. Supplemental Methods

**Study Population Criteria**

A diagnosis of liver cirrhosis was based on a combination of International Classification of Diseases Codes 10th Edition (ICD-10) diagnostic codes and laboratory values. patients had to meet ≥2 of the following criteria: a diagnosis of underlying liver disease, a diagnosis of portal hypertensive liver disease sequelae, or evidence of liver dysfunction on laboratory testing:

| **Criteria 1: Underlying Liver Disease** | **ICD-10 Codes** |
|---|---|
| Liver Cirrhosis (unspecified) | K71, K72, K73, K74, K75, and K77 |
| Viral Cirrhosis | B15, B16, B17, B18, and B19 |
| Alcoholic Cirrhosis | K70 |
| Nonalcoholic Steatohepatitis | K75.81, K76 |
| Portal Hypertensive Liver Disease | K76.6 |
| **Criteria 2: Portal Hypertensive Liver Disease Sequelae** | **ICD-10 Codes** |
| Esophageal Varices | I85, K22.89, and I86.4 |
| Spontaneous Bacterial Peritonitis | K65 |
| Hepatic Encephalopathy | K72.9, K72.91, G93.41, G93.4, B15.0, G92.8, G92.9, K72.11, B15.9, B16.0, B16.2, B16.9, B17.11, B19.0, B19.11, B19.21, K70.11, K70.31, K70.41, K71.11, K71.51, K72.01 |
| Hepatorenal Syndrome | K76.7 and K91.83 |
| **Criteria 3: Laboratory Evidence of Liver Dysfunction** | **Laboratory Thresholds** |
| Albumin | ≤ 3 g/dL |
| Total Bilirubin | ≥ 2mg/dL |
| International Normalized Ratio | ≥ 1.2 |

Inclusion criteria for study population (eligible patients met at least **two out of three** criteria listed). Laboratory values are within at least six months preceding right heart catheterization test.

**Comorbidity Criteria**

A diagnosis of left heart failure was based on a combination of ICD-10 diagnostic codes at the time of RHC and outpatient medication usage within 90 days of the index RHC. Patients had to meet **both of the following** criteria:

| Criteria 1: Diagnosis of Left Heart Failure | ICD-10 Codes |
|---|---|
| Left Ventricular Failure | I50.1 |
| Systolic Heart Failure | I50.2, I50.20, I50.21, I50.22, I50.23 |
| Diastolic Heart Failure | I50.3, I50.30, I50.31, I50.32, I50.33 |
| Combined Heart Failure | I50.4, I50.41, I50.42, I50.43 |
| **Criteria 2: Outpatient medication use within 90 days of index RHC** | **Qualifying Medications** |
| Beta-adrenergic receptor antagonist therapy | Carvedilol, Metoprolol, Bisoprolol, Propranolol, Labetalol |
| Sodium-glucose Cotransporter-2 (SGLT2) Inhibitor | Dapagliflozin, Empagliflozin, Canagliflozin |
| Angiotensin Converting Enzyme Inhibitor (ACE-I) or Angiotensin Receptor Blocker (ARB) | Lisinopril, Ramipril, Enalapril, Captopril, Losartan, Candesartan, Valsartan |
| Angiotensin Receptor Neprilysin Inhibitor (ARNI) | Sacubitril-Valsartan |

## Covariate Calculations

The CI was calculated by dividing the CO by the body surface area using the Du Bois formula[1-2], or directly obtained from the CART application if the calculation was unavailable. Thermodilution measures of cardiac function (CO and CI) were preferred for analyses, and estimated Fick was used only when thermodilution was unavailable. The PVR was calculated[1-2] as follows: $PVR = \frac{(mPAP-PAWP)}{CO}$. If not directly input, mPAP was calculated as follows: $mPAP = \frac{pulmonary\ arterial\ systolic\ pressure+(2*pulmonary\ arterial\ diastolic\ pressure)}{3}$.

The Model for End-Stage Liver Disease score (MELD) was calculated[26-27] as MELD = $10 * \left(0.957 * ln(Serum\ Creatinine) + (0.378 * ln(Bilirubin)) + (1.12 * ln(INR))\right) + 6.43$, the Sodium-adjusted MELD (MELD-Na) was calculated as MELD-Na = $MELD + 1.32 * (137 - Serum\ Sodium) - (0.033 * MELD * (137 - Serum\ Sodium))$, and the MELD excluding INR (MELD-XI) = $\left((11.76 * ln(Serum\ Creatinine) + (0.511 * ln(Bilirubin)) + 9.44\ )\right)$. For bilirubin and creatinine values <1 mg/dL, a value of 1.0 mg/dLwas used to avoid negative scores. For serum sodium values greater than 137 mEq/L, a value of 137 mEq/L was used, to avoid negative scores. Calculated MELD, MELD-Na, and MELD-XI were rounded to the nearest integer for analysis.

## Medication Definitions

Prescriptions for endothelin receptor antagonist therapy included bosentan, ambrisentan, or macitentan. Prescriptions for soluble guanylate cyclase stimulator therapy included riociguat. Prescriptions for prostacyclin pathway modulator therapy included selexipag, epoprostenol, or treprostinil. Prescriptions for phosphodiesterase-5 inhibitor therapy included sildenafil or tadalafil. Outpatient prescription fill of systemic anticoagulation therapy included warfarin, heparin, rivaroxaban, apixiban, edoxaban, dabigatran, or betrixaban. Outpatient prescription fill of liver cirrhosis therapy included rifaximin, propranolol, nadolol, lactulose, or ursodiol. Outpatient prescription fill of beta-adrenergic receptor antagonist therapy included carvedilol, metoprolol, bisoprolol, propranolol, and labetalol.

## Table S1: Cardiopulmonary hemodynamic characteristics of subjects in the study cohort

| Hemodynamic Variable | Median | $25^{th}$ Percentile | $75^{th}$ Percentile |
|---|---|---|---|
| **mPAP (mmHg)** | 30 | 23 | 38 |
| **PVR (WU)** | 2.0 | 1.3 | 3.2 |
| **CI (L/min/$m^2$)** | 2.4 | 1.9 | 3.0 |
| **PAWP (mmHg)** | 18 | 12 | 25 |

mPAP, mean pulmonary arterial pressure; PVR, pulmonary vascular resistance; PAWP, pulmonary artery wedge pressure; CI, cardiac index

## Table S2: Summary of hazard ratios for adjusted models between cardiopulmonary hemodynamic variables and all-cause mortality

| Clinical Variable | 1 YEAR MORTALITY | | | 3 YEAR MORTALITY | | |
|---|---|---|---|---|---|---|
| | Adjusted Hazard Ratio | 95% Confidence Interval | p-value | Adjusted Hazard Ratio | 95% Confidence Interval | p-value |
| **mPAP (mmHg)** | | | | | | |
| **28** | 1.28 | [0.87, 1.87] | 0.21 | 1.41 | [1.03, 1.93] | 0.03 |
| **30** | 1.41 | [0.97, 2.03] | 0.07 | 1.52 | [1.12, 2.06] | < 0.01 |
| **32** | 1.49 | [1.04, 2.14] | 0.03 | 1.58 | [1.17, 2.12] | < 0.01 |
| **34** | 1.53 | [1.07, 2.19] | 0.02 | 1.59 | [1.18, 2.14] | < 0.01 |
| **36** | 1.55 | [1.08, 2.23] | 0.02 | 1.59 | [1.17, 2.14] | < 0.01 |
| **PVR (Wood Units)** | | | | | | |
| **1.2** | 1.02 | [1.01, 1.04] | < 0.01 | 1.02 | [1.01, 1.03] | < 0.01 |
| **1.4** | 1.05 | [1.02, 1.07] | < 0.01 | 1.04 | [1.02, 1.06] | < 0.01 |
| **1.6** | 1.07 | [1.03, 1.11] | < 0.01 | 1.06 | [1.03, 1.10] | < 0.01 |
| **PAWP (mmHg)** | | | | | | |
| **12** | 1.01 | [0.97, 1.05] | 0.55 | 1.02 | [0.99, 1.06] | 0.20 |
| **15** | 1.13 | [1.01, 1.26] | 0.03 | 1.15 | [1.05, 1.26] | < 0.01 |
| **18** | 1.29 | [1.07, 1.54] | < 0.01 | 1.31 | [1.13, 1.52] | < 0.01 |

The reference mean pulmonary arterial pressure (mPAP) is 10 mmHg. The reference pulmonary vascular resistance (PVR) is 1 Wood Units. The reference pulmonary artery wedge pressure (PAWP) is 10 mmHg. Multivariable models were adjusted for age, sex, race, ethnicity, Veteran's Administration procedural site, MELD-XI, and comorbidities

**Table S3: Summary of hazard ratios for adjusted models between cardiac index and all-cause mortality**

| Cardiac Index ($L/min/m^2$) | 1 YEAR MORTALITY | | | 3 YEAR MORTALITY | | |
|---|---|---|---|---|---|---|
| | Adjusted Hazard Ratio | 95% Confidence Interval | p-value | Adjusted Hazard Ratio | 95% Confidence Interval | p-value |
| **1.0** | 2.24 | [1.71, 2.93] | < 0.01 | 1.93 | [1.51, 2.45] | < 0.01 |
| **1.5** | 1.80 | [1.52, 2.13] | < 0.01 | 1.56 | [1.36, 1.79] | < 0.01 |
| **2.0** | 1.31 | [1.16, 1.49] | < 0.01 | 1.21 | [1.09, 1.34] | < 0.01 |
| **3.0** | 1.06 | [0.97, 1.15] | 0.18 | 1.08 | [1.01, 1.16] | 0.03 |
| **3.5** | 1.17 | [1.00, 1.38] | 0.06 | 1.20 | [1.05, 1.37] | < 0.01 |
| **4.0** | 1.27 | [1.03, 1.56] | 0.03 | 1.29 | [1.08, 1.53] | < 0.01 |

The reference cardiac index (CI) value is 2.5 $L/min/m^2$. Multivariable models were adjusted for age, sex, race, ethnicity, Veteran's Administration procedural site, MELD-XI, and comorbidities.

**Table S4: Summary of hazard ratios for adjusted models between cardiac index and all-cause mortality, further adjusted for mPAP and PAWP**

| Cardiac Index ($L/min/m^2$) | 1 YEAR MORTALITY | | | 3 YEAR MORTALITY | | |
|---|---|---|---|---|---|---|
| | Adjusted Hazard Ratio | 95% Confidence Interval | p-value | Adjusted Hazard Ratio | 95% Confidence Interval | p-value |
| **1.0** | 1.94 | [1.47, 2.55] | < 0.01 | 1.69 | [1.32, 2.17] | < 0.01 |
| **1.5** | 1.68 | [1.41, 1.98] | < 0.01 | 1.46 | [1.27, 1.69] | < 0.01 |
| **2.0** | 1.28 | [1.13, 1.45] | < 0.01 | 1.18 | [1.07, 1.32] | < 0.01 |
| **3.0** | 1.07 | [0.98, 1.16] | 0.13 | 1.08 | [1.01, 1.16] | 0.03 |
| **3.5** | 1.19 | [1.01, 1.39] | 0.04 | 1.20 | [1.05, 1.37] | < 0.01 |
| **4.0** | 1.29 | [1.05, 1.59] | 0.02 | 1.30 | [1.09, 1.54] | < 0.01 |

The reference cardiac index (CI) value is 2.5 $L/min/m^2$. Multivariable models were adjusted for age, sex, race, ethnicity, Veteran's Administration procedural site, MELD-XI, and comorbidities.

## Table S5: Summary of hazard ratios for unadjusted and adjusted models between cardiopulmonary hemodynamics and 3-year mortality

| Clinical Variable | UNADJUSTED 3 YEAR MORTALITY | | | ADJUSTED 3 YEAR MORTALITY | | |
|---|---|---|---|---|---|---|
| | Hazard Ratio | 95% Confidence Interval | p-value | Hazard Ratio | 95% Confidence Interval | p-value |
| **mPAP (mmHg)** | | | | | | |
| **28** | 1.72 | [1.27, 2.34] | < 0.01 | 1.41 | [1.03, 1.93] | 0.03 |
| **30** | 1.89 | [1.40, 2.53] | < 0.01 | 1.52 | [1.12, 2.06] | < 0.01 |
| **32** | 1.99 | [1.49, 2.66] | < 0.01 | 1.58 | [1.17, 2.12] | < 0.01 |
| **PVR (Wood Units)** | | | | | | |
| **1.2** | 1.03 | [1.02, 1.04] | < 0.01 | 1.02 | [1.01, 1.03] | < 0.01 |
| **1.4** | 1.06 | [1.04, 1.09] | < 0.01 | 1.04 | [1.02, 1.06] | < 0.01 |
| **1.6** | 1.10 | [1.06, 1.13] | < 0.01 | 1.06 | [1.03, 1.10] | < 0.01 |
| **PAWP (mmHg)** | | | | | | |
| **12** | 1.03 | [1.00, 1.06] | 0.09 | 1.02 | [0.99, 1.06] | 0.20 |
| **15** | 1.17 | [1.07, 1.27] | < 0.01 | 1.15 | [1.05, 1.26] | < 0.01 |
| **18** | 1.35 | [1.17, 1.56] | < 0.01 | 1.31 | [1.13, 1.52] | < 0.01 |
| **CI (L/min/m$^2$)** | | | | | | |
| **1.0** | 1.89 | [1.49, 2.39] | < 0.01 | 1.93 | [1.51, 2.45] | < 0.01 |
| **2.0** | 1.25 | [1.12, 1.38] | < 0.01 | 1.21 | [1.09, 1.34] | < 0.01 |
| **3.0** | 1.14 | [1.06, 1.22] | < 0.01 | 1.08 | [1.01, 1.16] | 0.03 |
| **4.0** | 1.43 | [1.21, 1.69] | < 0.01 | 1.29 | [1.08, 1.53] | < 0.01 |

The reference mean pulmonary arterial pressure (mPAP) is 10 mmHg. The reference pulmonary vascular resistance (PVR) is 1 Wood Units. The reference pulmonary artery wedge pressure (PAWP) is 10 mmHg. The reference cardiac index (CI) value is 2.5 L/min/m$^2$. Multivariable models were adjusted for age, sex, race, ethnicity, Veteran's Administration procedural site, MELD-XI, and comorbidities.

**Table S6: Summary of hazard ratios for adjusted models between cardiopulmonary hemodynamics and all-cause mortality for patients with MELD-XI ≥12**

| Clinical Variable | 1 YEAR MORTALITY | | | 3 YEAR MORTALITY | | |
|---|---|---|---|---|---|---|
| | Adjusted Hazard Ratio | 95% Confidence Interval | p-value | Adjusted Hazard Ratio | 95% Confidence Interval | p-value |
| **mPAP (mmHg)** | | | | | | |
| **28** | 1.48 | [0.92, 2.38] | 0.11 | 1.77 | [1.17, 2.68] | < 0.01 |
| **30** | 1.65 | [1.04, 2.61] | 0.03 | 1.92 | [1.29, 2.87] | < 0.01 |
| **32** | 1.79 | [1.15, 2.80] | 0.01 | 2.03 | [1.38, 3.00] | < 0.01 |
| **34** | 1.87 | [1.20, 2.91] | < 0.01 | 2.09 | [1.42, 3.07] | < 0.01 |
| **36** | 1.92 | [1.23, 2.99] | < 0.01 | 2.11 | [1.43, 3.12] | < 0.01 |
| **PVR (Wood Units)** | | | | | | |
| **1.2** | 1.02 | [1.01, 1.04] | < 0.01 | 1.02 | [1.01, 1.03] | < 0.01 |
| **1.4** | 1.05 | [1.01, 1.08] | < 0.01 | 1.04 | [1.01, 1.07] | < 0.01 |
| **1.6** | 1.07 | [1.02, 1.11] | < 0.01 | 1.06 | [1.02, 1.10] | < 0.01 |
| **PAWP (mmHg)** | | | | | | |
| **12** | 1.00 | [0.95, 1.05] | 0.99 | 1.02 | [0.97, 1.06] | 0.51 |
| **15** | 1.12 | [1.01, 1.24] | 0.03 | 1.13 | [1.03, 1.24] | < 0.01 |
| **18** | 1.35 | [1.12, 1.63] | < 0.01 | 1.33 | [1.14, 1.56] | < 0.01 |
| **CI (L/min/$m^2$)** | | | | | | |
| **1.0** | 2.27 | [1.69, 3.05] | < 0.01 | 1.84 | [1.40, 2.41] | < 0.01 |
| **1.5** | 1.75 | [1.45, 2.11] | < 0.01 | 1.58 | [1.34, 1.85] | < 0.01 |
| **2.0** | 1.25 | [1.09, 1.42] | < 0.01 | 1.21 | [1.08, 1.35] | < 0.01 |
| **3.0** | 1.14 | [1.04, 1.25] | < 0.01 | 1.15 | [1.06, 1.24] | < 0.01 |
| **3.5** | 1.33 | [1.11, 1.58] | < 0.01 | 1.35 | [1.17, 1.57] | < 0.01 |
| **4.0** | 1.48 | [1.18, 1.85] | < 0.01 | 1.51 | [1.25, 1.82] | < 0.01 |

The reference mean pulmonary arterial pressure (mPAP) is 10 mmHg. The reference pulmonary vascular resistance (PVR) is 1 Wood Units. The reference pulmonary artery wedge pressure (PAWP) is 10 mmHg. The reference cardiac index (CI) value is 2.5 L/min/$m^2$. Multivariable models were adjusted for age, sex, race, ethnicity, Veteran's Administration procedural site, MELD-XI, and comorbidities

**Table S7: Summary of hazard ratios for adjusted models between cardiopulmonary hemodynamics and all-cause mortality for patients with MELD-XI <12**

| Clinical Variable | 1 YEAR MORTALITY | | | 3 YEAR MORTALITY | | |
|---|---|---|---|---|---|---|
| | Adjusted Hazard Ratio | 95% Confidence Interval | p-value | Adjusted Hazard Ratio | 95% Confidence Interval | p-value |
| **mPAP (mmHg)** | | | | | | |
| **28** | 0.95 | [0.50, 1.79] | 0.87 | 0.96 | [0.60, 1.54] | 0.86 |
| **30** | 0.99 | [0.54, 1.86] | 0.99 | 0.98 | [0.61, 1.56] | 0.92 |
| **32** | 1.02 | [0.55, 1.90] | 0.95 | 0.95 | [0.6, 1.52] | 0.84 |
| **34** | 1.04 | [0.55, 1.95] | 0.91 | 0.92 | [0.57, 1.48] | 0.72 |
| **36** | 1.06 | [0.55, 2.03] | 0.86 | 0.90 | [0.55, 1.47] | 0.67 |
| **PVR (Wood Units)** | | | | | | |
| **1.2** | 1.02 | [0.98, 1.05] | 0.37 | 1.02 | [0.99, 1.04] | 0.23 |
| **1.4** | 1.03 | [0.97, 1.10] | 0.34 | 1.03 | [0.98, 1.09] | 0.22 |
| **1.6** | 1.05 | [0.96, 1.15] | 0.32 | 1.05 | [0.97, 1.13] | 0.20 |
| **PAWP (mmHg)** | | | | | | |
| **12** | 0.98 | [0.89, 1.09] | 0.76 | 1.03 | [0.95, 1.11] | 0.51 |
| **15** | 1.04 | [0.76, 1.43] | 0.81 | 1.19 | [0.93, 1.51] | 0.17 |
| **18** | 1.13 | [0.75, 1.68] | 0.56 | 1.30 | [0.96, 1.76] | 0.09 |
| **CI (L/min/m$^2$)** | | | | | | |
| **1.0** | 2.60 | [1.25, 5.38] | 0.01 | 3.18 | [1.76, 5.72] | < 0.01 |
| **1.5** | 1.76 | [1.18, 2.62] | < 0.01 | 1.52 | [1.12, 2.07] | < 0.01 |
| **2.0** | 1.25 | [0.90, 1.72] | 0.18 | 0.99 | [0.78, 1.29] | 0.99 |
| **3.0** | 1.09 | [0.86, 1.38] | 0.48 | 1.10 | [0.92, 1.32] | 0.30 |
| **3.5** | 1.26 | [0.82, 1.92] | 0.29 | 1.21 | [0.88, 1.66] | 0.25 |
| **4.0** | 1.45 | [0.88, 2.39] | 0.15 | 1.32 | [0.90, 1.93] | 0.15 |

The reference mean pulmonary arterial pressure (mPAP) is 10 mmHg. The reference pulmonary vascular resistance (PVR) is 1 Wood Units. The reference pulmonary artery wedge pressure (PAWP) is 10 mmHg. The reference cardiac index (CI) value is 2.5 L/min/m$^2$. Multivariable models were adjusted for age, sex, race, ethnicity, Veteran's Administration procedural site, MELD-XI, and comorbidities

## Table S8: Adjusted survival probabilities at 6 months, 1-year, and 3-years by subgroup

| | 6 Months | | 1 Year | | 3 Years | |
|---|---|---|---|---|---|---|
| **Subgroups** | **Survival Probability** | **95% CI** | **Survival Probability** | **95% CI** | **Survival Probability** | **95% CI** |
| **Hyperdynamic Circulation** | 0.87 | [0.84,0.90] | 0.79 | [0.75,0.83] | 0.58 | [0.53,0.64] |
| **Post-capillary PH** | 0.76 | [0.75,0.78] | 0.67 | [0.65,0.69] | 0.44 | [0.42,0.46] |
| **PoPH** | 0.77 | [0.73,0.81] | 0.63 | [0.59,0.68] | 0.42 | [0.37,0.48] |
| **Non-PH Cirrhosis** | 0.89 | [0.87,0.92] | 0.82 | [0.79,0.85] | 0.62 | [0.58,0.67] |

Non-PH Cirrhosis, no pulmonary hypertension (mPAP ≤20mmHg) at the time of right heart catheterization; Hyperdynamic Circulation, pulmonary hypertension in cirrhosis (mPAP >20mmHg) without elevations in pulmonary artery wedge pressure (PAWP ≤15mmHg) or pulmonary vascular resistance (PVR <3 WU); Post-capillary PH, pulmonary hypertension in cirrhosis with an elevated pulmonary artery wedge pressure (PAWP >15mmHg); PoPH, pulmonary hypertension in cirrhosis with a precapillary pattern (PVR ≥3 WU, PAWP ≤15mmHg). Survival probabilities with 95% confidence intervals at six months, 1-year, and 3-years stratified by subgroups (Hyperdynamic Circulation, Post-capillary PH, PoPH, Non-PH cirrhosis).

**Figure S1** Histogram distribution of cardiopulmonary hemodynamic values in the study cohort

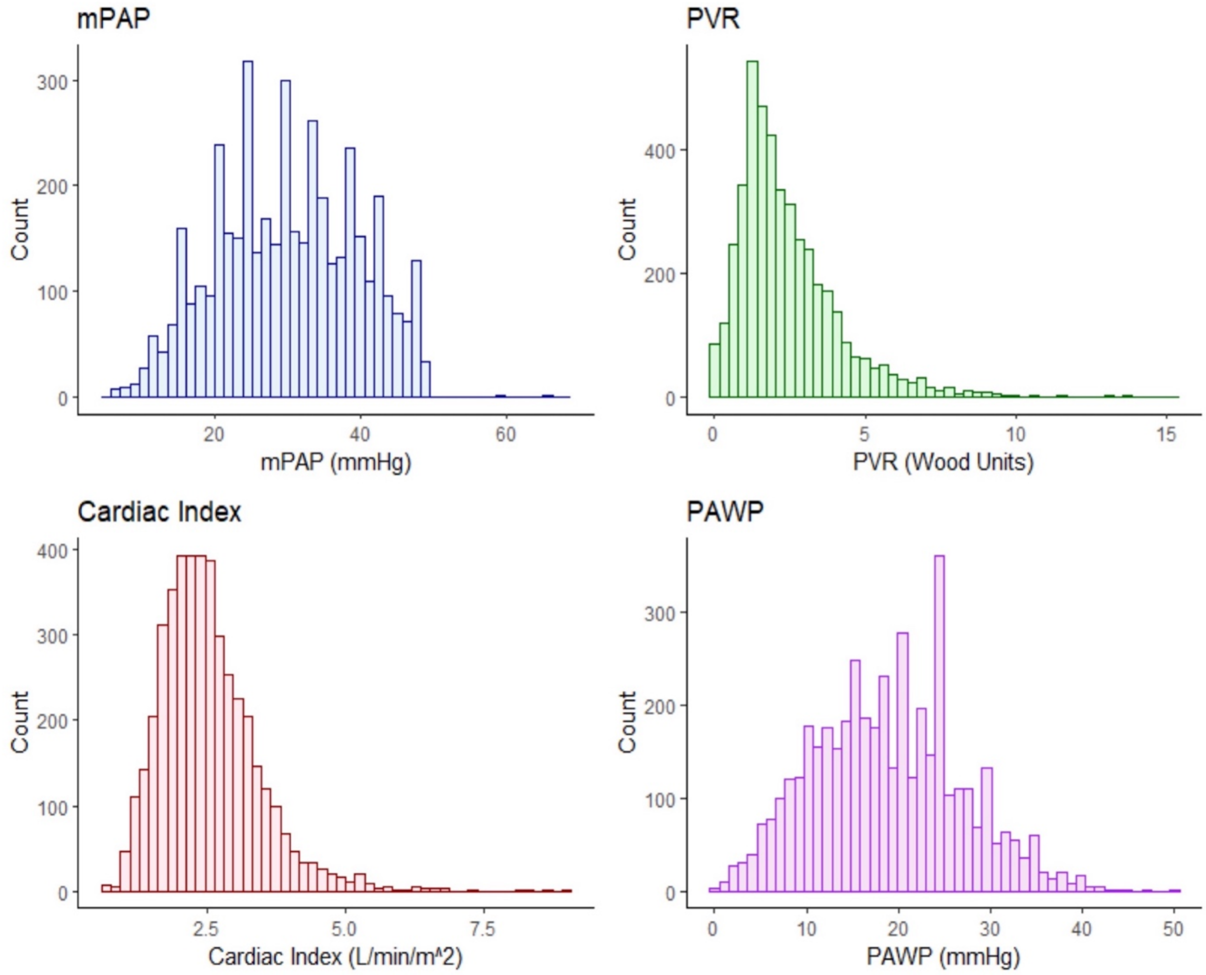


Histograms depicting the distributions of mean pulmonary arterial pressure (mPAP), pulmonary vascular resistance (PVR), cardiac index (CI), and pulmonary artery wedge pressure (PAWP) for the full cohort.

**Figure S2** The association between PVR and mortality in the study cohort

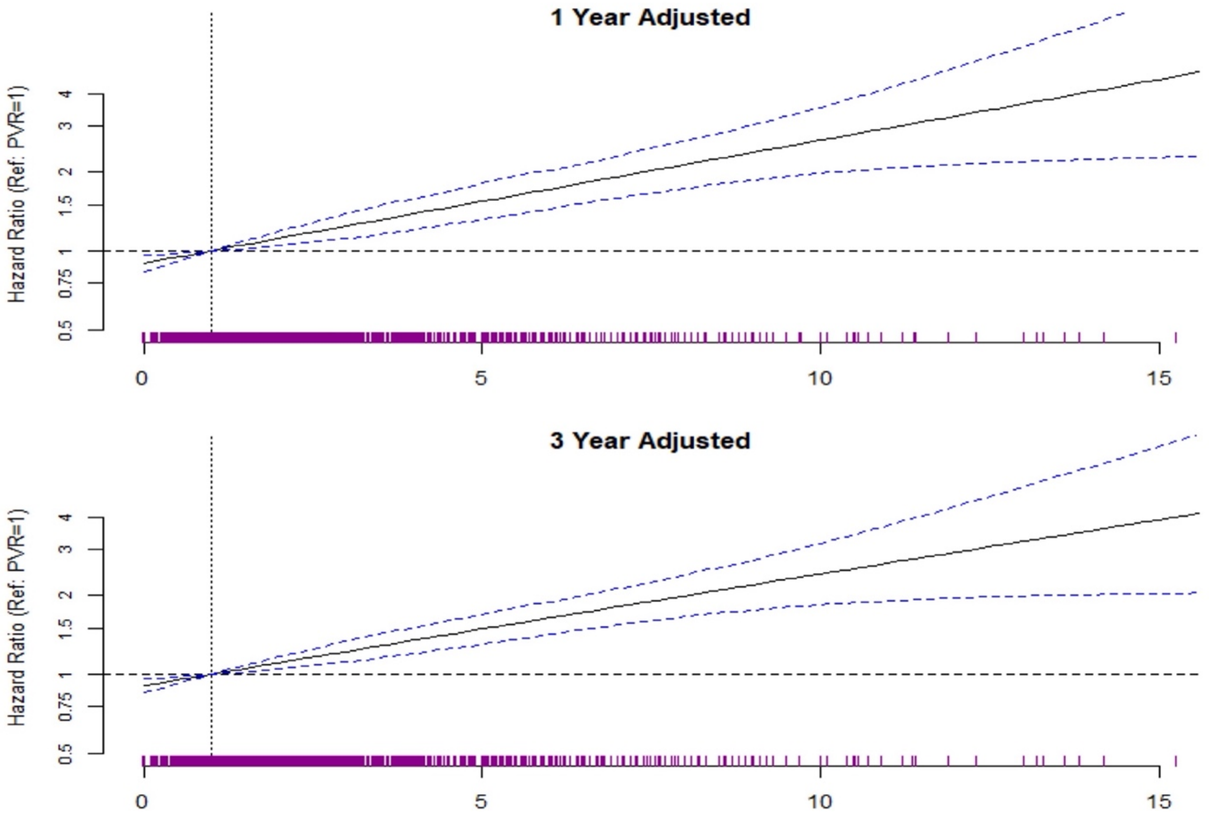


Adjusted hazard ratio for all-cause mortality at 1- and 3-years following right heart catheterization as a function of pulmonary vascular resistance (PVR). Hazard ratios (solid line) with 95% confidence intervals (dashed bands) are plotted for PVR relative to a reference value of 1 WU (vertical dotted line). The hazard ratios on the y-axis are demarcated by a logarithmic scale, and a hazard ratio of 1 is depicted by a horizontal dotted line. A rugplot showing the distribution of PVR values is displayed directly above the x-axis. Multivariable models adjusted for age, sex, race, ethnicity, Veteran's Administration procedural site, MELD-XI, and comorbidities.

**Figure S3** The association between PAWP and mortality in the study cohort

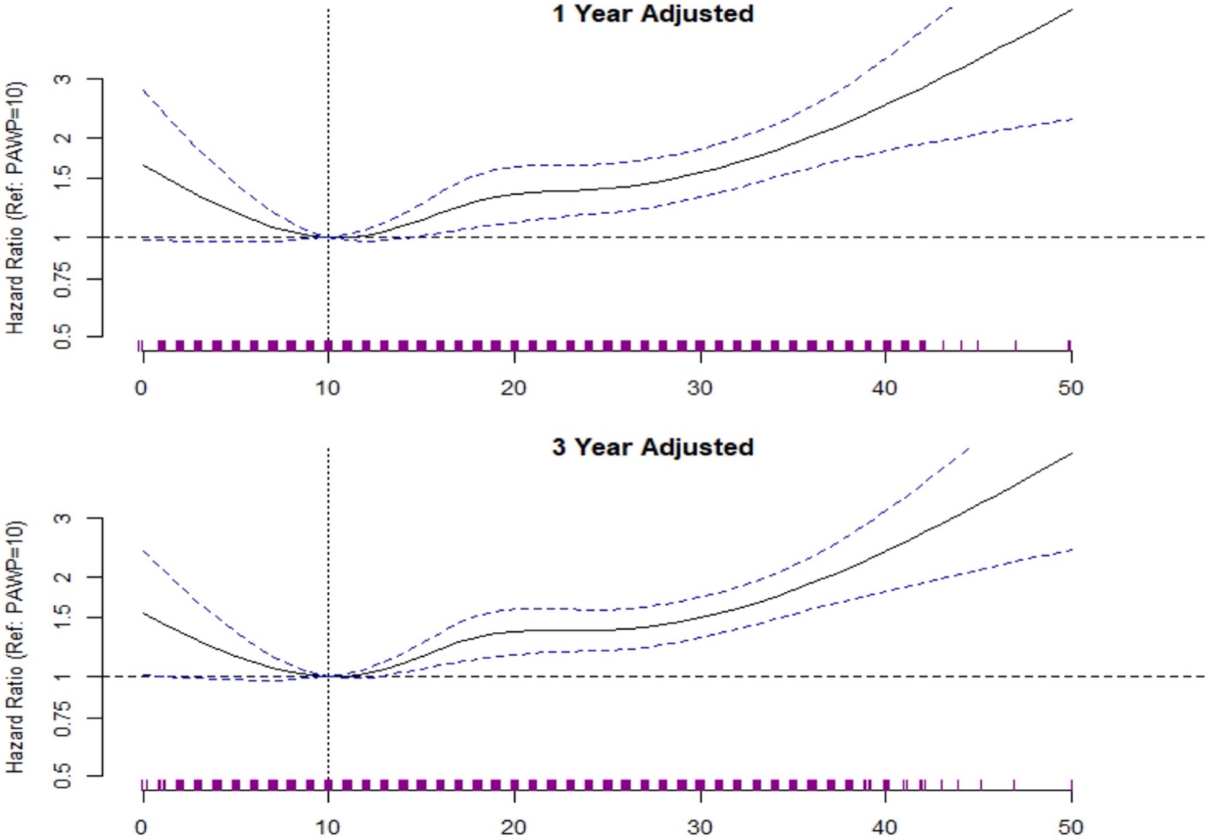


Adjusted hazard ratio for all-cause mortality at 1- and 3-years following right heart catheterization as a function of pulmonary artery wedge pressure (PAWP). Hazard ratios (solid line) with 95% confidence intervals (dashed bands) are plotted for PAWP relative to a reference value of 10 mmHg (vertical dotted line). The hazard ratios on the y-axis are demarcated by a logarithmic scale, and a hazard ratio of 1 is depicted by a horizontal dotted line. A rugplot showing the distribution of PAWP values is displayed directly above the x-axis. Multivariable models adjusted for age, sex, race, ethnicity, Veteran's Administration procedural site, MELD-XI, and comorbidities

**Figure S4** Kaplan Meier analysis for survival probability dichotomized by CI

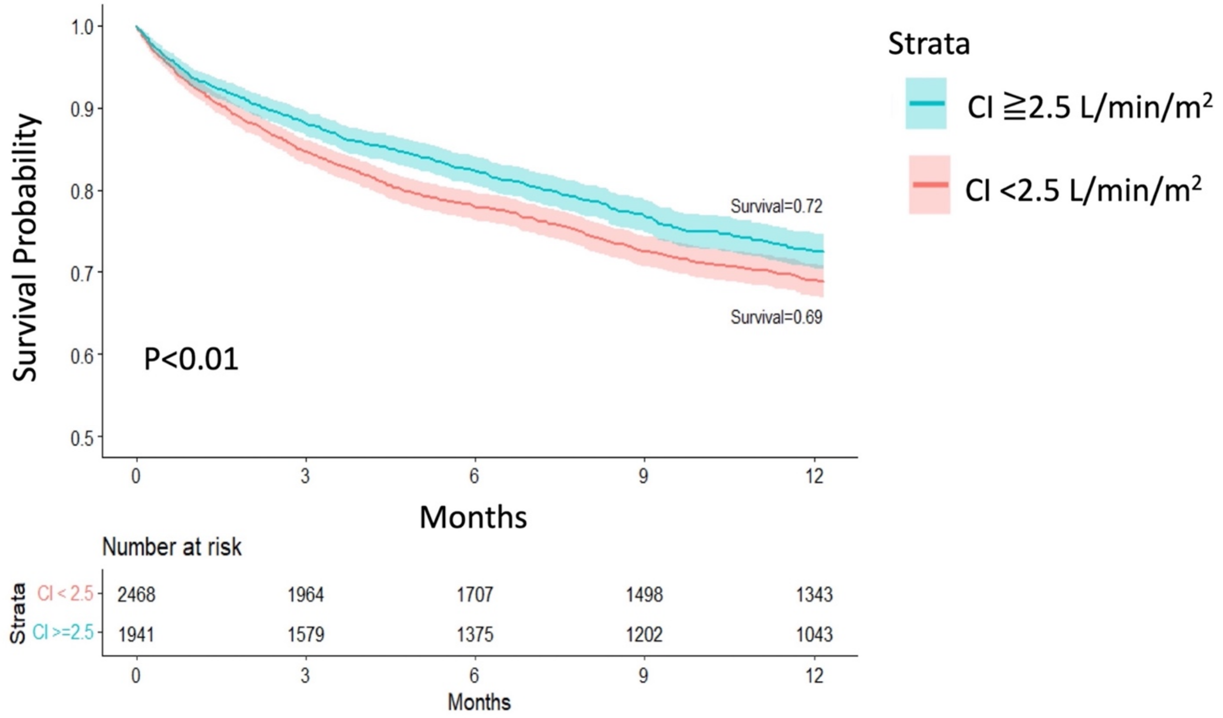


Kaplan-Meier analysis for the patient cohort dichotomized by CI. Green, high CI (≥2.5 L/min/$m^2$); red, low CI (<2.5 L/min/$m^2$). Log-rank test p-value displayed.

**Figure S5** Kaplan Meier analysis for survival probability in PoPH patients dichotomized by PAH-targeted therapy

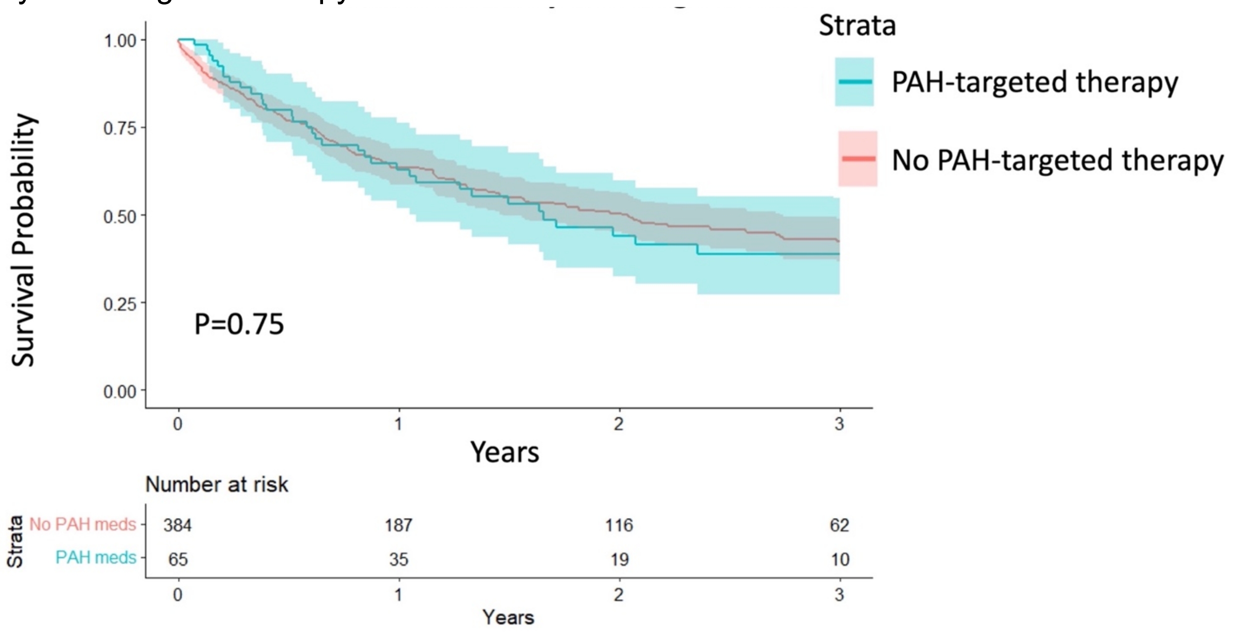


Kaplan-Meier analysis for the portopulmonary hypertension (PoPH) subgroup dichotomized by treatment with PAH-targeted therapy. Blue, treated with PAH-targeted therapy; red, no use of PAH-targeted therapy. Log-rank test p-value is displayed.
